# Bias in RNA-seq Library Preparation: Current Challenges and Solutions

**DOI:** 10.1155/2021/6647597

**Published:** 2021-04-19

**Authors:** Huajuan Shi, Ying Zhou, Erteng Jia, Min Pan, Yunfei Bai, Qinyu Ge

**Affiliations:** ^1^State Key Laboratory of Bioelectronics, School of Biological Science and Medical Engineering, Southeast University, Nanjing 210096, China; ^2^School of Medicine, Southeast University, Nanjing 210097, China

## Abstract

Although RNA sequencing (RNA-seq) has become the most advanced technology for transcriptome analysis, it also confronts various challenges. As we all know, the workflow of RNA-seq is extremely complicated and it is easy to produce bias. This may damage the quality of RNA-seq dataset and lead to an incorrect interpretation for sequencing result. Thus, our detailed understanding of the source and nature of these biases is essential for the interpretation of RNA-seq data, finding methods to improve the quality of RNA-seq experimental, or development bioinformatics tools to compensate for these biases. Here, we discuss the sources of experimental bias in RNA-seq. And for each type of bias, we discussed the method for improvement, in order to provide some useful suggestions for researcher in RNA-seq experimental.

## 1. Introduction

With the development of massive parallel sequencing, high-throughput sequencing (NGS) of RNA (RNA-seq) has become a very common tool in molecular biology. It almost affects our understanding for the function of genomic [1] and provides valuable resources for other scientific disciplines. However, RNA-seq is a process of extremely intricate, including RNA extraction and purification, library construction, sequencing, and bioinformatics analysis. These processes can inevitably introduce some deviations (Table 1), which influence the quality of RNA-seq datasets and result in their erroneous interpretation. Therefore, understanding these biases is critical to avoiding erroneous interpretation of the data and to realize the full potential of this powerful technology.

**Figure 1:**
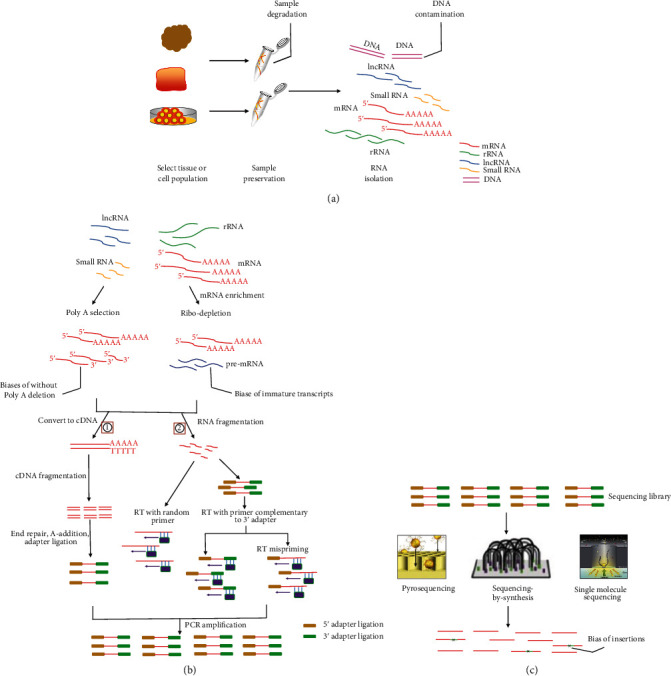
Simplified protocol of RNA-seq experiment and sources of bias. (a) Sample preservation and isolation. These biases can include sample degradation, DNA contamination. (b) Strategies for cDNA library construction. ①: the RNA directly converts to cDNA; then, cDNA was fragmented and library preparation. ②: classical a protocol. One method involves reverse transcription (RT) using random primers first, subsequently adapter ligations and sequencing (left). The other method is to first sequentially ligate 3′ and 5′ adapters, followed by performing cDNA synthesis with a primer complementary to the adapter (RT-primer), subsequently sequencing (right). On using the RT primer with a specific sequence, mispriming could occur due to annealing of the RT-primer to transcript sequences with some complementarity (RT mispriming). (c) RNA-seq platform (including Pyrosequencing, sequencing-by-synthesis, and single-molecule sequencing). These biases can be introduced by insertions and deletions, raw single-pass data, etc.

Generally, the representative workflow of RNA-seq analysis includes the extraction and purification of RNA from cell or tissue, the preparation of sequencing library, including fragmentation, linear or PCR amplification, RNA sequencing, and the processing and analysis of sequencing data (Figure 1). Commonly used NGS platforms, including Illumina and Pacific Biosciences, need PCR amplification during library construction to increase the number of cDNA molecules to meet the needs of sequencing. Nevertheless, the most problematic step in sample preparation procedures is amplification. It is due to the fact that PCR amplification stochastically introduces biases, which can propagate to later cycles [2]. In addition, PCR also amplifies different molecules with unequal probabilities, leading to the uneven amplification of cDNA molecules [3, 4]. Recently, researchers have proposed several different methods in order to reduce PCR amplification, such as PCR-free protocols and isothermal amplification. Nevertheless, these methods are not perfect and still present some artifacts and biases of sequencing. Consequently, understanding these biases is critical to get reliable data and will provide some useful advice to the researcher.

In this perspective article, we summarize the current situation and solutions on biases and discuss the source of bias in RNA-seq. The key point will be on solutions to reduce bias and improve the quality of library sequencing platform. Furthermore, we highlight the bias sources of different methods of amplification and how can amplification bias be reduced.

## 2. Sample Preservation and Isolation

Despite many studies have shown that RNA-seq has many advantages, it is still a rapidly developing biotechnology and faces several challenges. Among them, one often overlooked aspect is the sample preparation process, which may also bring potential variations and deviations on RNA-seq experiment, including RNA isolation, sample processing, library storage time, RNA input level (such as the difference in the number of start-up RNA), and sample cryopreservation (such as fresh or frozen preservation). Generally, good preservation of sample that may be used for transcriptome studies is more important, because many transcriptome protocols require high-quantity and high-quality nucleic acids [5]. Therefore, we will discuss the bias of sample in different preservation and isolation methods. A sum up and improvement suggestions are shown in Table 2.

### 2.1. The Storage and Preservation Methods of RNA

Studies have been demonstrated that RNA degradation is closely related to sample preservation or fixation method. At the present, as far as we know, the standard storage of tissues for RNA-seq has been in liquid nitrogen or freeze stored at -80°C. Unfortunately, frozen specimens are not widely available because they are costly to collect and maintain. Therefore, in diagnostic pathology archives, most tissue samples rely on the formalin-fixed and paraffin-embedded (FFPE) method for preservation [6]. Nevertheless, nucleic acids are more difficult to extract from FFPE tissue, because of the need to remove paraffin and counteract the covalent protein DNA interaction during the fixation process [7, 8]. Additionally, fixation delay, fixation process, tissue preparation, paraffin embedding, and archival preservation may lead to fragmentation, cross-linking, and chemical modification of FFPE tissue-derived nucleic acids, resulting in poor sequencing libraries [9]. Recently, the researcher proposed an optimization scheme [9]; the main problem to be considered with this method is as follows: (1) by minimizing the sample processing and freezing and thawing cycles, ensure that RNA is preserved as best as possible after extraction; (2) for degradation samples, it is best to use high sample input; (3) in the reverse transcription step, use random priming instead of oligo-dT or specific sequence as primers. These suggestions might help to mitigate some sequencing biases or errors to some extent, so that we can make full use of the FFPE sample to obtain reliable results.

### 2.2. The Isolation and Extraction of RNA

High-quality RNA purification is the premise of RNA-seq. However, due to the widespread existence of RNA degrading enzymes (RNases) [10–12], successful isolation of high-quality RNA remains challenging. At the present, the RNA extraction method can be divided into two types, including TRIzol (phenol: chloroform extraction) and Qiagen (silica-gel-based column procedures). These methods were mainly developed to extract long mRNAs and have been based on the assumption that all RNAs are equally purified, when these methods are applied to noncoding RNAs, which may be resulted in RNA degradation [13]. Currently, the mirVana kit was reported to be the best tool for producing high-yield and high-quality RNA [14].

## 3. Library Construction

After RNA isolation and extraction, the next step is library construction of transcriptome sequencing. Library construction usually begins with the depletion of ribosomal RNA (rRNA) or the enrichment of mRNA enrichment, because most of the total RNA of cellular or tissue is rRNA. For eukaryotic transcriptome, polyadenylated mRNAs are usually extracted by oligo-dT beads, or rRNAs are selectively depleted. Unlikely, prokaryote mRNAs are not stably polyadenylated. Hence, oligo d(T)-mediated messenger enrichment is not suitable; there is only the second option. Then, RNA is usually fragmented to a certain size range by physical or chemical method. The subsequent steps differ among experimental design and NGS platforms. However, studies indicated that most of the protocols currently used for library construction may introduce serious deviations. For example, RNA fragmentation can introduce length biases or errors, subsequently propagating to later cycles. Furthermore, library amplification may also be affected by primer bias, such as primer bias in multiple displacement amplification (MDA) [15], primer mismatch in PCR amplification [16, 17]. As a consequence, it may introduce nonlinear effects and inevitably compromise the quality of RNA-seq dataset, leading to the result of erroneous interpretation. Consequently, in the next section, we will describe and summarize the bias sources of library preparation, including mRNA enrichment, fragmentation, primer bias, adapter ligation, reverse transcription, and especially PCR. A sum up suggestions for improvement is presented in Table 3.

### 3.1. Input RNA

Notwithstanding, RNA-seq can be used to measure transcripts of any sample in principle; it has been a challenge to apply standard protocols to samples with either very low quantity or low quality (partially degraded) input RNA. It is due to the fact that the bias associated with low amounts of input RNA has strong and harmful effects on downstream analysis. If not noticed, this may have significant impact on the subsequent biological interpretation. Recently, the researcher has proposed several different methods to overcome the challenges of low-quality or low-quantity RNA sample, including RNase H (also known as SDRNA) [18], Ribo-Zero [19], SMART4 [20], Ovation RNA-seq System (NuGEN) [21], and Duplex-Specific Nuclease (DSN) light normalization [22]. Adiconis et al. [23] compared the relative merits of each method with a standard high-input and high-quality sample group, determining its applicability for a specific project. This result showed that RNase H was the best method for detecting low-quality RNA and even could effectively replace the standard RNA-seq method based on oligo (dT). For low-quantity RNA, the SMART and NuGEN approaches had lower duplication rates and significantly decreased the necessary amount of starting material compared to other methods.

### 3.2. rRNA Depletion

rRNAs are very abundant, often constituting 80% to 90% of total RNA. Due to the fact that rRNA sequence rarely arouse people's interest in RNA-seq experiments, it is necessary to remove rRNA from sample before library construction. The aim is in order to prevent most of the library and the majority of sequencing reads from being rRNA. A standard solution is to enrich for the polyadenylated (poly (A)) RNA transcripts with oligo (dT) primers. For eukaryotes, studies have been shown that oligo (dT) provides technical convenience for enriching mRNA from sample; it is due to the fact that most mRNA and many long noncoding RNAs (lncRNAs) have poly (A) tail [24]. Nevertheless, in addition to rRNA, this method also removes all non-poly (A) RNAs, such as replication-dependent histones, various lncRNAs, and bacterial mRNA [25, 26]. Moreover, oligo (dT) is difficult to capture incomplete mRNA molecules (such as mRNAs lacking intact poly (A) tails). Therefore, if the starting materials are from the FFPE sample, it is not the best method, because the RNAs of FFPE were degraded to a small average size. On the other hand, the non-poly (A) tailed mRNA enrichment method can be used to isolate RNA from any eukaryotic organism [27].

The second rRNA depletion approach takes the opposite method, targeting rRNA molecules and removing them. This rRNA depletion method can be used for subsequent sequencing of all non-rRNA molecules and is not limited to complete mRNA molecules. Unlike the oligo (dT), rRNA depletion relies on the exact sequence content of the ribosomal RNA, so commercially available or each given kits will only be effective for a specific group of species whose rRNA sequences complement the probes in the kit [28]. However, because rRNA depletion depends on sequence-specific hybridization of probe, there is a risk of nonspecific cross-hybridization and transcript removal, leading to biased representation of that transcript in the sequencing data. The researcher gave two suggestions to the selection of kit, oligonucleotide probes and an antibody specific for RNA: DNA hybrids, to minimize the effects of the oligonucleotide mishybridization [28]. On the other hand, rRNA depletion may capture more immature transcripts, leading to a complexity increase of sequencing data [29]. However, neither method can enrich poly (A) transcripts, such as poly (A)-histone mRNAs, including histone H1 variants [27]. Moreover, the rRNA depletion method is remarkably more expensive than mRNA isolation.

### 3.3. RNA Fragmentation

Currently, RNA is usually fragmented due to read length restriction (<600 bp) of sequencing technologies and the sensitivity of amplification to long cDNA molecules. There are two major approaches of RNA fragmentation: chemical (using metal ions) and enzymatic (using RNase III) [30]. Commonly, RNA is fragmented using metal ions such as Mg++ and Zn++ in high temperatures and alkaline conditions. This method yields more accurate transcript identification than RNase III digestion [31]. This result was also confirmed in Wery et al. [31]. Furthermore, intact RNAs can be reverse transcribed (RT) to cDNA by reverse transcriptase, subsequently was fragmented. Then, the cDNA was fragmented using the enzymatic or physical method. Examples of the enzymatic method include DNase I digestion, nonspecific endonuclease (like NEBNext dsDNA Fragmentase from New England Biolabs), and transposase-mediated DNA fragmentation (Illumina Nextera XT). However, the Tn5 transposase method showed sequence-specific bias [32], which is the preferred method when only small quantities of cDNA are available, since the cDNA fragmentation and adapter ligation are connected in one step [33]. Studies have shown that nonspecific restriction endonucleases indicate less sequence bias and have been shown to perform similarly to the physical methods with respect to cleavage-site sequence bias and coverage uniformity of target DNA [34, 35]. Another advantage of the enzymatic method is that they are easy to automate [36]. The physical method includes acoustic shearing, sonication, and hydrodynamic [17, 37, 38], which also can present nonrandom DNA fragmentation bias [35]. However, the physical cDNA fragmentation method is less amenable to automation than RNA fragmentation. Therefore, the physical method will be replaced by commercially available kits and the enzymatic method.

### 3.4. Primer Bias

Commonly, after mRNA is fragmented, which can be reverse transcribed into cDNA by random hexamers. However, studies have been indicated that random hexamer primer can lead to the deviation of nucleotide content of RNA sequencing reads, which also affects the consistency of the locations of reads along expressed transcripts. This may result in low complexity of RNA sequencing data. Given this bias, Mamanova et al. [39] proposed an alternative to RNA-seq using the Illumina Genome Analyzer. The reverse transcription takes place directly on the flowcells which yield stranded reads and avoids the amplification of polymerase chain reaction. RNA is not transformed into dscDNA using random priming but directly connected to RNA fragment by sequencing adapters. Then, the ligated RNA library is reverse transcribed on the flowcells [40]. Thus, the deviation is avoided due to primer. In addition, the researcher proposed using a bioinformatics tool, via reweighing scheme to adjust for the bias and make the distribution of the reads more uniform.

### 3.5. Adapter Ligation

Generally, as for the deep sequencing of RNA library preparation, a critical step is the ligation of adapter sequences. The selection of T4 RNA ligase (Rnl1 or Rnl2) or other RNA ligase is very important. Subsequently, the ligation products were amplified by PCR. Or, nucleotide homopolymer sequences were added by poly (A) polymerase [41] or terminal deoxyribonucleotidyl transferase [41] but prevent the unambiguous determination of the termini of the input RNAs. This method has also been widely used in the construction of small RNA library. Recently, studies have shown that adapter ligation introduces a significant but widely overlooked bias in the results of NGS small RNA sequencing. Hence, in order to alleviate this bias, the new Bio Scientific NEXTflex V2 protocol uses a set of random nucleotide adapters at the ligation boundary. And the study indicated that this protocol can reliably detect several Illumina-based methods to evade the capture of miRNAs. Although these results did not show a clear standard for small RNA library preparation, the data of the NEXTflex protocol had the best correlation with RT-qPCR [42].

### 3.6. Reverse Transcription

Currently, the strategies of transcriptome analysis are still to convert RNA to cDNA before sequencing. A known feature of reverse transcriptases is that they tend to produce false second strand cDNA through DNA-dependent DNA polymerase. This may not be able to distinguish the sense and antisense transcript and create difficulties for the data analysis. The researcher proposed several modifications. Among them, the deoxyuridine triphosphate (dUTP) method, one of the leading cDNA-based strategies, can be specifically removed by enzymatic digestion [43]. It can provide excellent library complexity, chain specificity, coverage uniformity, consistency with known annotation, and accuracy for expression analysis [44]. However, the effectiveness of antisense transcription near highly expressed genes should be carefully measured, since a small number of reads (about 1%) have been observed on the opposite chain [45]. Another method is to synthesize the first strand of cDNA using labeled random hexamer primer and SSS using DNA-RNA template-switching primer. Nevertheless, the two methods are laborious [46]. Additionally, for the SSS method, it requires a nonstandard sequencing data analysis scheme, and as part of the genome, complexity is lost in the process of converting four bases into three bases; about 30% of the unique matching sequencing readings are lost. Furthermore, the combination of random primers and template switching may lead to uneven gene coverage.

### 3.7. PCR Amplification

PCR is a basic tool widely in molecular biology laboratories. In particular, the combination of PCR and NGS sequencing promoted the explosive development of RNA sequence acquisition. However, PCR amplification has been proved to be the main source of artifacts and base composition bias in the process of library construction, which may lead to misleading or inaccurate conclusions in data analysis. Therefore, it is essential to avoid PCR bias, and great efforts have been expended on trying to control and mitigate bias in current. In the next section, we will discuss the sources of bias in PCR amplification and suggestions for improvement.

### 3.8. The Sources of PCR Amplification Biases and Improvement Methods

#### 3.8.1. Extremely AT/GC-Rich

Studies have been indicated that fragments of GC-neutral can be amplified more than GC-rich or AT-rich fragments. Therefore, the fragments with high AT or very high GC content often have little or no amplification at all [47, 48]. These unfavorable features result in difficulties in genome sequencing of extremely AT-rich, such as human malaria parasite [48], or high GC (Bordetella pertussis) genomes (average GC content, about 75%). Bearing this in mind, Aird et al. [17] present a protocol to reduces the introduction of GC bias in the PCR library preparation stage by switching the polymerases, prolonging the denaturation step, and reducing the annealing and extension temperature. On the other hand, researchers developed a without amplification library construction approach [39]. Through the use of custom adapters, the samples without amplification and ligation can be hybridized directly with the oligonucleotides on the flowcell surface, thus avoiding the biases and duplicates of PCR. However, the amplification-free method requires high sample input, which limits its widely used.

Besides, the Phusion polymerase method is commonly used in PCR amplification at present, compared with other polymerase methods, which have processivity and fidelity [49]. However, the amplification efficiency of extremely (G+C)-rich or (A+T)-rich fragments was lower efficiency than (G+C)-neutral fragments. Several laboratories have compared different PCR polymerases and conditions to minimize amplification bias. For example, Quail et al. [49] estimated a large series of polymerases. This result showed that the best total enzyme was Kapa HiFi (Kapa Biosystems), because the genome coverage with Kapa HiFi was more uniform than Phusion, which was very close to the result without PCR.

In addition, in order to overcome the amplification bias of AT/GC-rich, it is necessary to the addition of substances for enhancement PCR specificity and/or yield. The most effective PCR enhancing additives currently used are betaine [50]. It is an amino acid mimic that acts to balance the differential *T*_*m*_ between AT and GC base pairs and has been effectively used to improve the coverage of GC-rich templates [17]. Furthermore, Oyola et al. [48] tested various PCR amplification conditions by testing a series of polymerases and their tolerance to AT-rich templates in the absence or presence of tetramethylammonium chloride (TMAC). Their result showed that the TMAC can remarkably increase the amplification of AT-rich regions in Kapa HiFi in the presence. Additionally, a number of additives have been reported to play an important role in reducing the bias of PCR amplification, including small amides such as formamide, small sulfoxides such as dimethyl sulfoxide (DMSO), or reducing compounds such as *β*-mercaptoethanol or dithiothreitol (DTT) [50].

#### 3.8.2. PCR Cycle

As we all know, PCR can exponentially amplify DNA/cDNA templates, thus leading to a significant increase of amplification bias with the number of PCR cycles [51]. Therefore, it is recommended that PCR be performed using as few cycle numbers as possible to mitigation bias [52, 53]. At the present, several laboratories have compared different PCR cycle number to reduce amplification bias. Wu et al. [54] performed a comprehensive analysis. The results of the study indicate that comparing with the lower cycle number, the higher cycle number can produce significant biases or artifacts in standard amplifications of mixed templates. In addition, Sze and Schloss's [55] study indicated that reducing the number of cycles of amplification can also decrease PCR biases and artifacts using a mock community and human stool samples.

### 3.9. Alternative Methods for Library Amplification

Although PCR amplification has many advantages, it also has some disadvantages. For example, time-consuming thermal cycling is needed to obtain the target sequence amplification at different temperatures, which led to the development of alternative amplification methods [56, 57]. Among them, isothermal amplification does not need any thermal cycle, which is easier to operate than PCR, and requires less energy. These characteristics greatly simplify the realization of isothermal amplification of diagnostic equipment in medical points. In 2006, Piepenburg and colleagues proposed new methods of recombinase polymerase amplification (RPA), which characteristic of the reaction is a constant temperature by a strand-displacement polymerase [58]. And the sensitivity of RPA is similar compared to PCR. Another method is linear amplification for deep sequencing (LADS), which connects the two different sequencing adapters to the blunt end repair and A-tailed fragments; then, one of them is extended with the sequence of T7 RNA polymerase promoter to form a linearly amplified library [59]. Compared to standard PCR-amplified libraries, T7-amplified libraries can remarkably reduce the bias of AT- or GC-rich, yet strand information is not maintained. Accordingly, if directionality needs to be maintained, it must either be introduced before adapter ligation or it requires modification of the LADS protocol. For instance, during the synthesis of the first strand of cDNA, the “barcode” can be incorporated on the antistrand and double-stranded cDNA can be generated to start LADS.

At present, the preparation of genomic from clinical samples is still a bottleneck in sequencing analysis and frequently limits by the amount of specimen available. Therefore, the amplification of samples is indispensable to get sufficient sample yield in sequencing. Researchers proposed a whole-genome amplification (WGA) method, which can generate a large amount of DNA/cDNA directly from small cell samples. Subsequently, the entire genome fragments were amplified by multiple displacement amplification (MDA) in 30°C condition, which uses *φ*29 DNA polymerase and random exonuclease-resistant primers [60]. In addition, Dean et al.'s [60] study showed that MDA could generate large quantities and high-quality cDNA directly from the starting material. For this reason, MDA became an optimal choice for WGA from single cells.

Additionally, what is worth mentioning here is that an amplification-free RNA-seq protocol has been reported [39]. In the method, the ligating adapters contain primers annealing and attachment to the flowcell surface, subsequently the amplification step of a standard cluster. Hence, the PCR amplification step is avoided during library preparation and efficiently tackles this problem of PCR amplification bias. Nevertheless, when only a limited amount of starting material is available, the method is inappropriate, because the amplification-free method needs several hundred nanograms of input sample for library preparation [47].

## 4. Sequencing and Imaging

It is very important for the selection of sequencing platform in RNA-seq experiment. Currently, commercially available NGS platforms include Illumina/Solexa Genome Analyser, Life Technologies/ABI SOLiD System, and Roche/454 Genome Sequencer FLX [61]. These platforms use a sequencing-by-synthesis approach to sort tens of millions of sequence clusters in parallel. Generally, the NGS platform can be classified as either ensemble-based (sequencing multiple identical copies of a DNA molecule) or monomolecular (sequencing a single DNA molecule). Nevertheless, studies have found that sequencing technologies often have systematic defects. For example, when the wrong bases are introduced in the process of template cloning and amplification, substitution bias may appear in platforms such as Illumina and SOLiD®, which limits the utility of data. In addition, studies pointed out that sequence-specific bias may be caused by single-strand DNA folding or sequence-specific changes in enzyme preference [62]. Pacific Biosciences SMRT platform produces long single molecular sequences that are vulnerable to misinsertion from nonfluorescent nucleotides [63, 64]. Besides, the sequencing platform can produce representative biases, that is, some base composition regions (especially those with very high or very low GC composition) are not fully represented, thus leading to bias in the results [65]. Consequently, we will briefly discuss the bias of sequencing platforms, mainly including the Illumina and single-molecule-based platforms. A sum up of suggestions for improvement is presented in Table 4.

Currently, the Illumina HiSeq platform is the most widely used next-generation RNA sequencing technology and has become the standard of NGS sequencing. The platform has two flowcells, each of which provides eight separate channels for sequencing reaction. The sequencing reaction takes 1.5 to 12 days to complete, depending on the total read length of the library. Minoche et al.'s [66] study discovered that the HiSeq platform exists error types of GC content bias. In addition, Illumina released the MiSeq, which integrates NGS instruments and provides end-to-end sequencing solutions using reversible terminator sequencing-by-synthesis technology. The MiSeq instrument is a desktop classifier with low throughput but faster turnaround (generating about 30 million paired-end reads in 24 h). Simultaneously, it can perform on-board cluster generation, amplification, and data analysis in a single run, including base calls, alignment, and variant calling. At the present, MiSeq has become a dominant platform for gene amplification and sequencing in microbial ecology. Nevertheless, various technical problems still remain, such as reproducibility, hence hampered harnessing its true potential to sequence. Furthermore, Fadrosh et al.'s [67] study found that MiSeq 16S rRNA gene amplicon sequencing may arise “low sequence diversity” problems in the first several cycles.

Furthermore, the emergence of single-molecule sequencing platforms such as PacBio makes single-molecule real-time (SMRT) sequencing possible [68]. In this method, DNA polymerase and fluorescent-labeled nucleoside were used for uninterrupted template-directed synthesis. One advantage of SMRT is that it does not include the PCR amplification step, as a consequence avoiding amplification bias. At the same time, this sequencing approach can produce extraordinarily long reads with average lengths of 4200 to 8500 bp, which greatly improves the detection of new transcriptional structures [69, 70], in addition, due to the relatively low cost per run of PacBio's, which can reduce the cost of RNA-seq. However, PacBio can usually introduce high error rates (∼5%) compared to Illumina and 454 sequencing platform [71]. Due to the fact that it is difficult to the matching erroneous reads to the reference genome, thus the high error rate may be lead to misalignment and loss of sequencing reads. Furthermore, Fichot and Norman's [72] study showed that PacBio's sequencing platform can shun enrichment bias of extremely GC/AT.

## 5. Discussion and Conclusion

At the present, RNA-seq has been widely used in biological, medical, clinical, and pharmaceutical research. However, all these sequencing studies are limited by the accuracy of underlying sequencing experiments, because RNA-seq technology may introduce various errors and biases in sample preparation, library construction, sequencing and imaging, etc.

It is well known that RNA is extremely labile and degradable. Therefore, if the sample cannot be separated immediately after collection, which can be kept in intermediary solution. At the present, RNAlater (Thermo Fisher Scientific and Qiagen) and RNAstable (Sigma-Aldrich) are commonly used stabilizers, which can prevent RNA degradation and maintain RNA integrity. Additionally, the extraction and isolation of RNA have been proved as one of the most bias sources. Currently, TRIzol is a frequently used method. Furthermore, some protocols of RNA extraction and isolation may carry over some gDNA into total RNA samples, which can be removed by DNA enzyme treatment to prevention gDNA contamination (false positive signal).

Additionally, library construction methods are frequently biased, which is a main concern for RNA-seq data quality. Among them, PCR amplification is the major source of bias. A previous study showed that GC content has a virtual influence on PCR amplification efficiency. Therefore, we suggest that Kapa HiFi (Kapa Biosystems, Wilmington, MA) or AccuPrime Taq DNA Polymerase High Fidelity (Life Technologies) can be selected to PCR amplification. It has been shown that these enzymes can minimize the amplification bias caused by the extreme GC content. Furthermore, Aird et al. [17] found that, for extremely high GC samples, the amplification bias can remarkably be reduced by modification of the denaturation time and subsequent PCR melt cycles. Moreover, the reduction number of amplification cycles can also improve PCR bias. Therefore, the methods of no-PCR amplification were developed, but these require a lot of input material [39], resulting in a limitation for a low input sample. Therefore, when the amount of input material is limited, amplification is indispensable.

In summary, the major goal of constructing the sequencing library is to minimize the bias. The bias was frequently defined as the systematic distortion of data due to the experimental protocols. Therefore, it is impossible to eliminate all sources of experimental bias. The best strategies are as follows: (i) to understand how the bias is generated and to take measures to minimize it; (ii) to pay attention to the experimental design and minimize the influence of irreducible bias on the final analysis.

## Figures and Tables

**Table 1:** Sources of main bias in RNA-seq.

Bias sources
Sample preservation (1) Degradation of RNA: such as tissue autolysis; nucleic acid degradation and cross-linking during the preparation of formalin-fixed; formalin-fixed paraffin-embedded (FFPE) [6] (2) RNA extraction: such as using TRIzol [12] (3) Alien sequence contamination [73] (4) Low-quality and/or low-quantity RNA [23]
Library preparation (1) mRNA enrichment bias: such as 3′-end capture bias [74] (2) RNA fragmentation bias [31] (3) Primer bias: such as random hexamer bias; mispriming; nonspecific binding [75] (4) Adapter ligation bias: such as adaptor contamination [41] (5) Reverse transcription bias [76] (6) PCR amplification bias [77] (7) Machine failure; for example, incorrect PCR cycling temperatures [17]
Sequencing and imaging (1) Experimenter bias: such as cluster crosstalk caused by overloading the flowcell [78] (2) Sequencing platform bias [65] (3) Sequence context: such as AT/GC enrichment [79] (4) Machine failure: such as failure of laser, hard drive, software, and fluidics

**Table 2:** Sources of bias in RNA-seq sample preservation and suggestions for improvement.

Description	Suggestion for improvement
Sample preservation	
FFPE methods: causes modifications of biomolecules, such as cross-linkage of nucleic acids with proteins	Use of non-cross-linking organic fixatives and methacarn solution [6]
RNA extraction	
Using TRIzol: small RNA loss at low concentrations	Use high concentrations of RNA samples or avoid TRIzol extraction altogether [80] Use alternative protocols such as the mirVana miRNA isolation kit [14]

**Table 3:** Sources of bias in RNA-seq library preparation and suggestions for improvement.

Description	Suggestion for improvement
mRNA enrichment	
3′-end capture bias that is introduced during poly (A) enrichment in RNA sequencing	Use rRNA depletion [81].
Fragmentation	
RNA fragmentation by RNase III: not completely random, leading to reduced complexity	Use chemical treatment (e.g., zinc) rather than RNase III for RNA fragmentation [31] Intact RNAs can be reverse transcribed to cDNA by reverse transcriptase, then which was fragmented by mechanical or enzymatic methods [82]
Priming bias	
Random hexamer priming bias	RNA is not converted to dscDNA using random priming, instead of sequencing adapters that are ligated directly onto RNA fragments [39] A read count reweighing scheme was proposed that adjusts for the bias and makes the distribution of reads more uniform [40]
Adapter ligation	
Adapter ligation bias: due to substrate preferences of T4 RNA ligases	Use adapters with random nucleotides at the extremities to be ligated [42]
PCR	
(1) Bias due to preferential amplification of with neutral GC% (2) The number of cycles of high PCR amplification	Use Kapa HiFi rather than Phusion polymerase [49] For extremely AT/GC-rich genomes, use the PCR additive TMAC or betaine, or lower extension temperatures and extended denaturation times [17, 48] Reduce the number of cycles of amplification [55] For the amplification of minute quantities of genomic DNA (single cell), use MDA rather than PCR [83] A large number of starting material, use amplification-free PCR [47]

**Table 4:** The bias sources of major sequencing platforms.

Company	Platforms	Sequencing	Dominant bias type	Suggestion for improvement
Roche/454 Life Sciences	GS FLX Titanium XL+	Pyrosequencing	The bias of sequencing was introduced by PCR amplification prior to sequencing.	Reduction of the number of PCR cycles and use of DNA polymerases with even higher fidelity [84]
	GS FLX Titanium XLR70			
	GS Junior			
	HiSeq 2000			

Illumina	Genome Analyzer IIx	Sequencing-by-synthesis with reversible terminator	Substitution type miscalls are the major source of bias.	Quality trimming (sickle) combined with error correction (BayesHammer) followed by read overlapping (PANDAseq) as the most suitable approach, reducing substitution biases [85]
	MiSeq			
	SOLiD™ 4 system			
	Ion PGM™ sequencer (318 chip)			

Helicos BioSciences	HeliScope™ single molecule sequencer	Single-molecule sequencing	Biases were introduced by insertions and deletions.	If a low sequencing bias is needed, Illumina or SOLiD are often the best choices [86, 87]

Pacific Biosciences	PacBio RS	Single-molecule sequencing	High bias of raw single-pass data	
